# Variability of *Symbiodinium* Communities in Waters, Sediments, and Corals of Thermally Distinct Reef Pools in American Samoa

**DOI:** 10.1371/journal.pone.0145099

**Published:** 2015-12-29

**Authors:** Ross Cunning, Denise M. Yost, Marisa L. Guarinello, Hollie M. Putnam, Ruth D. Gates

**Affiliations:** 1 University of Hawai‘i, Hawai‘i Institute of Marine Biology, PO Box 1346, Kāne‘ohe, Hawaii, 96744, United States of America; 2 Northwest Knowledge Network, University of Idaho, 875 Perimeter Dr. MS2358, Moscow, Idaho, 83844, United States of America; Biodiversity Research Center, Academia Sinica, TAIWAN

## Abstract

Reef-building corals host assemblages of symbiotic algae (*Symbiodinium* spp.) whose diversity and abundance may fluctuate under different conditions, potentially facilitating acclimatization to environmental change. The composition of free-living *Symbiodinium* in reef waters and sediments may also be environmentally labile and may influence symbiotic assemblages by mediating supply and dispersal. The magnitude and spatial scales of environmental influence over *Symbiodinium* composition in different reef habitat compartments are, however, not well understood. We used pyrosequencing to compare *Symbiodinium* in sediments, water, and ten coral species between two backreef pools in American Samoa with contrasting thermal environments. We found distinct compartmental assemblages of clades A, C, D, F, and/or G *Symbiodinium* types, with strong differences between pools in water, sediments, and two coral species. In the pool with higher and more variable temperatures, abundance of various clade A and C types differed compared to the other pool, while abundance of D types was lower in sediments but higher in water and in *Pavona venosa*, revealing an altered habitat distribution and potential linkages among compartments. The lack of between-pool effects in other coral species was due to either low overall variability (in the case of *Porites*) or high within-pool variability. *Symbiodinium* communities in water and sediment also showed within-pool structure, indicating that environmental influences may operate over multiple, small spatial scales. This work suggests that *Symbiodinium* composition is highly labile in reef waters, sediments, and some corals, but the underlying drivers and functional consequences of this plasticity require further testing with high spatial resolution biological and environmental sampling.

## Introduction

Coral reefs are among the most biologically diverse ecosystems on Earth and provide valuable ecosystem services as sources of tourism, coastal protection, natural products, primary productivity, and nutrition [1]. At the foundation of these ecosystems is the symbiosis between corals and diverse unicellular dinoflagellates in the genus *Symbiodinium*, which provide corals the nutrition they need to build calcium carbonate skeletons and accrete large reef structures [2]. Reefs are declining worldwide in large part due to the breakdown of this symbiosis (coral “bleaching” [3]) in response to environmental stressors, particularly high sea surface temperature anomalies, which are predicted to become more frequent and severe with climate change [4]. However, plasticity in corals’ symbiotic associations, i.e., their ability to associate with different *Symbiodinium* partners that are better adapted to different environmental conditions, may allow corals to acclimatize as conditions change [5,6]. Indeed, different *Symbiodinium* partners may alter the growth, energetics, and heat tolerance of their coral hosts [7,8]. Whether dynamic symbioses may realistically benefit corals under rapid climate change is unclear [9], in part due to a poor understanding of the ecological drivers of *Symbiodinium* community assembly and stability on reefs.

The composition of coral symbiont assemblages is influenced by both innate symbiotic specificity or flexibility [10,11] and environmental conditions at multiple spatial scales [12,13]. Contrasting patterns of stability or change in the dominant (i.e., most numerically abundant) symbiont type of different coral species across environmental gradients suggest that symbioses may be flexible in some corals but not others [14]. However, in addition to dominant symbionts, diverse lower-abundance taxa may also be present and dynamic, although they have been historically understudied due to methodological constraints [11,15]. More comprehensive and quantitative studies leveraging next-generation sequencing (NGS) are now emerging [16–20] and are necessary to understand how whole *Symbiodinium* assemblages are structured and influenced by the environment.

Symbiont community composition may also be influenced by *Symbiodinium* present in nearby hosts as well as in other reef habitat compartments, such as sediments and seawater. These free-living *Symbiodinium* communities generally are distinct from, but overlap with, endosymbiotic diversity [21–23], but have not yet been fully characterized by NGS. Linkages between free-living and symbiotic communities are expected, as *Symbiodinium* are routinely expelled by symbiotic hosts [24], actively dispersed by host larvae and corallivorous fishes [25–27], and exogenously acquired by both juvenile [28,29] and adult [30] hosts. Therefore, a metacommunity framework, in which an array of local communities are connected by dispersal [31], may provide insight into the drivers and scales of *Symbiodinium* community assembly on reefs [32]. In this context, it is also critical to understand how free-living communities in other reef habitat compartments are structured and influenced by the environment, which until now has received little study [23].

Here, we investigate environmental influence on *Symbiodinium* communities in reef waters, sediments, and ten coral species by comparing two backreef pools on Ofu Island, American Samoa. These pools are characterized by differences in size, depth, and water flow as well as different temperature ranges and extremes. Their contrasting thermal regimes and rich context of past environmental and ecological data [33–36] make these pools an ideal natural setting for this investigation. We utilized 454 pyrosequencing of the internal transcribed spacer 2 (ITS2) region to conduct a large-scale marker gene survey with high-sensitivity detection of rare taxa [17,37], representing the most comprehensive and quantitative study of both symbiotic and free-living *Symbiodinium* on reefs to date.

Limitations of this approach include 1) intragenomic sequence variation of the ITS2 marker [38] which decouples sequence diversity from biological and/or functional diversity, 2) copy number variation [39,40] which decouples sequence abundance from organismal abundance, and 3) quantitative biases introduced by nucleic acid extraction and PCR [41], which further separate sequence abundance data from true community composition. In light of these limitations, we take a conservative approach in identifying taxa, make no attempt at alpha diversity estimation, and make only relative between-pool comparisons of taxon abundance within compartments. Indeed, read abundance is approximately quantitative within taxa [41], and high quantitative accuracy of ITS2 pyrosequencing has been shown previously for *Symbiodinium* [17]. Nevertheless, the quantitative analyses employed here are performed on read abundance as a proxy for community composition in making relative comparisons within an ecological context, and should not be interpreted as evidence for specific taxon delimitation or abundance. With this approach, we provide new insight into the role of the environment in shaping *Symbiodinium* composition in reef habitats and suggest that a metacommunity framework may further advance the field.

## Materials and Methods

### *Symbiodinium* sampling

*Symbiodinium* communities associated with water, sediments, and corals were sampled in two backreef pools with highly (pool 300) and moderately (pool 400) variable temperature regimes on the southeast shore of Ofu Island, American Samoa, between December 2–14, 2011. GPS coordinates were recorded at each sample’s location, except for *Pocillopora damicornis* in pool 300 due to instrument failure. Seawater samples of 1 L were collected approximately 1 m below the surface at 1 m intervals along three transects parallel to and with increasing distance from shore in each pool (n = 28 in pool 300, n = 30 in pool 400). Sediment samples of 1 mL were collected with a syringe at 0.7–2 m depth throughout both pools (n = 30 each), then resuspended in 1 L of 0.2 μm-filtered seawater. Water and sediment samples were passed sequentially through 20 and 5 μm filters to capture particles between 5–20 μm diameter (including *Symbiodinium*), and 5 μm filters were placed directly into guanidinium DNA extraction buffer (50% w/v guanidinium isothiocyanate; 50 mM Tris pH 7.6; 10 μM EDTA; 4.2% w/v sarkosyl; 2.1% v/v β-mercaptoethanol). Colonies of ten coral species (*Psammacora contigua*, *Porites mound*, *Porites annae*, *Pocillopora damicornis*, *Pavona venosa*, *Leptoria phrygia*, *Goniastrea retiformis*, *Favia matthaii*, *Acropora pagoensis*, and *Acropora austera*), representing dominant coral taxa [33] were sampled between 0.3–2 m depth in each pool (n = 95 total; 2–6 per species per pool) by removing < 1 cm^2^ of skeleton and tissue from the colony surface with a small chisel. Coral samples were rinsed with deionized water and placed directly into DNA extraction buffer. All samples were transported to the Hawaii Institute of Marine Biology at the University of Hawaii for laboratory analysis.

### Amplicon sequencing

To extract genomic DNA, samples were incubated at 72°C for 20 min in guanidinium buffer (described above) and centrifuged at 16,000 x *g* for 5 min. Supernatant was mixed with an equal volume of isopropanol to precipitate DNA. Pellets were washed in 70% ethanol and resuspended in Tris buffer (0.1 M pH 8). To analyze *Symbiodinium* community structure, amplicon libraries of the internal transcribed spacer (ITS2) region of nuclear ribosomal DNA were prepared for pyrosequencing. The ITS2 locus was amplified in 25 μL PCR reactions containing 1× buffer, 2 mM MgCl_2_, 0.1 mM each dNTP, 0.1 μM biotinylated ‘its-dino’ primer, 0.1 μM ‘its2rev2’ [42] primer ligated with sample-specific 8 bp tag sequences, and 2 μL template DNA, diluted as necessary to achieve amplification. Thermal cycling began with 10 min at 95°C followed by 35 cycles of 30 s at 95°C, 40 s at 54°C, and 60 s at 72°C, with a final extension step of 10 min at 72°C. PCR products were pooled into two separate libraries and sequenced on a Roche GS-FLX system by Research and Testing Laboratory, LLC (Lubbock, TX).

### Bioinformatic analysis

Raw data for each sequencing run were processed through the standard analysis pipeline of Research and Testing Laboratory LLC, consisting of 1) trimming read ends where the running quality score average falls below Q = 25, 2) dereplication and removal of singletons > 4% divergent from other sequences, 3) *de novo* chimera checking and removal, and 4) denoising of base pair errors and poor reads [43]. Prior to analysis, sequences were demultiplexed based on their unique barcodes and sequences with homopolymer regions > 6 bases were discarded. Forward and reverse primer sequences were trimmed using *cutadapt* [44], allowing 3 indels/mismatches, and sequences without a forward primer match were discarded. Sequences were assigned taxonomy using SymTyper (Belcaid et al., in revision; [19]; www.symtyper.com), a custom bioinformatic pipeline developed for *Symbiodinium* ITS2 sequences, and a custom reference database of 719 *Symbiodinium* sequences manually obtained from GenBank (see Dryad data archive, doi:10.5061/dryad.32md8). SymTyper assigned sequences to the *Symbiodinium* clade level using a Hidden Markov Model approach, with clade assignment based on an e-value cutoff of 10^−20^ and a ratio of 10^−5^ relative to the next best clade hit. Sequences successfully classified at the clade level were then assigned to the subtype level by basic local alignment search tool (BLAST) with an e-value cutoff of 10^−20^ and a length-adaptive similarity threshold of > 97% over > 90% of the reference sequence length (Belcaid et al., in revision). Sequences that equally matched multiple references were phylogenetically placed at the internal tree node of the matches’ lowest common ancestor and assigned names in the format “<clade>_i:<node number>”. Sequences that did not pass subtype assignment cutoffs were classified as “new” and subsequently clustered *de novo* at 97% similarity in QIIME [45] using the *uclust* algorithm. 97% clustering was chosen as it was recently shown to appropriately classify sequences generated from isoclonal *Symbiodinium* lineages [20]. Each *de novo* cluster was assigned a name in the format “<clade>_d:<cluster number>” using the clade assigned by SymTyper. The closest BLAST hits for *de novo* representative sequences and closest leaf taxa for internal nodes are provided in S1 Table. Read counts of *Symbiodinium* taxa from SymTyper and *de novo* clustering were merged into a single table for downstream analysis.

### Statistical analysis

Taxa count data and associated metadata were imported into the R statistical computing environment [46] using the package *phyloseq* [47], and subset by compartment (i.e., coral, water, or sediment) and coral species for downstream analyses. Bray-Curtis dissimilarities among samples were calculated from relative abundance data that were square-root transformed to reflect differences in both dominant and non-dominant taxa. Dissimilarity matrices were then used to generate non-metric multidimensional scaling (NMDS) biplots and test for differences between pools in each compartment and species by one-way permutational multivariate analysis of variance (PERMANOVA) using the *adonis* function in the *vegan* package [48]. These one-way tests followed a two-way test with a significant interaction term, and a multiple testing correction was not used since less than one type I error would be expected. Following a significant one-way pool effect, *Symbiodinium* taxa that were differentially abundant between pools were identified using Bayesian Poisson-lognormal generalized linear mixed models in the R package *MCMC*.*OTU* [49], using raw count data as input. Only taxa that comprised > 0.1% of all sequences within a compartment or species were tested for differential abundance to reduce the number of tests performed. Models were fit for each compartment and coral species with pool as a fixed factor and abundances of each taxon in each pool were calculated relative to a modeled artificial taxon representing the sum of all counts, which controls for differences in sequencing depth among samples and factor levels [16]. Fold-changes in abundance between pools were calculated for each *Symbiodinium* taxon and significantly differentially abundant taxa were identified based on a Bayesian z-score p-values with a false discovery rate (FDR) of 0.1. Within-pool spatial autocorrelation for each compartment and coral species was analyzed using a Mantel test comparing Euclidean distance (based on GPS coordinates) and Bray-Curtis dissimilarity in the R package *ecodist* [50]. Circular plots displaying differential abundance were created using the R package *circlize* [51]. Georectified aerial images of the pools were projected in universal transverse mercator coordinates and plotted with sample GPS locations in R using the *raster* package [52]. All data and scripts to reproduce the analyses and figures in this paper are available at Dryad (doi:10.5061/dryad.32md8).

## Results

### Amplicon sequencing and bioinformatic analysis

After initial quality filtering, demultiplexing, and trimming, the dataset contained a total of 1,090,214 sequences. The SymTyper pipeline assigned 380,476 sequences to 421 *Symbiodinium* taxa and identified 59,293 putatively “new” *Symbiodinium* sequences that subsequently clustered into 1,186 *de novo* OTUs at 97% similarity. The number of *Symbiodinium* sequences per sample followed a log-normal distribution ranging from 33 to 19,201 (water: 210–19,201; sediment: 39–8,472; coral: 33–9,960) with an overall geometric mean of 1,011 ± 3.8 (gsd) (S1 and S3 Figs). Total read counts for individual taxa ranged from 1 to 157,687 with a distribution typical of microbial communities with many rare taxa [37] (S1 Fig). *Symbiodinium* taxa represented by internal nodes (i.e., “X_i:X”) or *de novo* clusters (i.e., “X_d:X”) are contextualized among taxa presently in the database by identifying the closest leaf taxon or closest BLAST hit, respectively (S1 Table).

### *Symbiodinium* community composition

**Fig 1:**
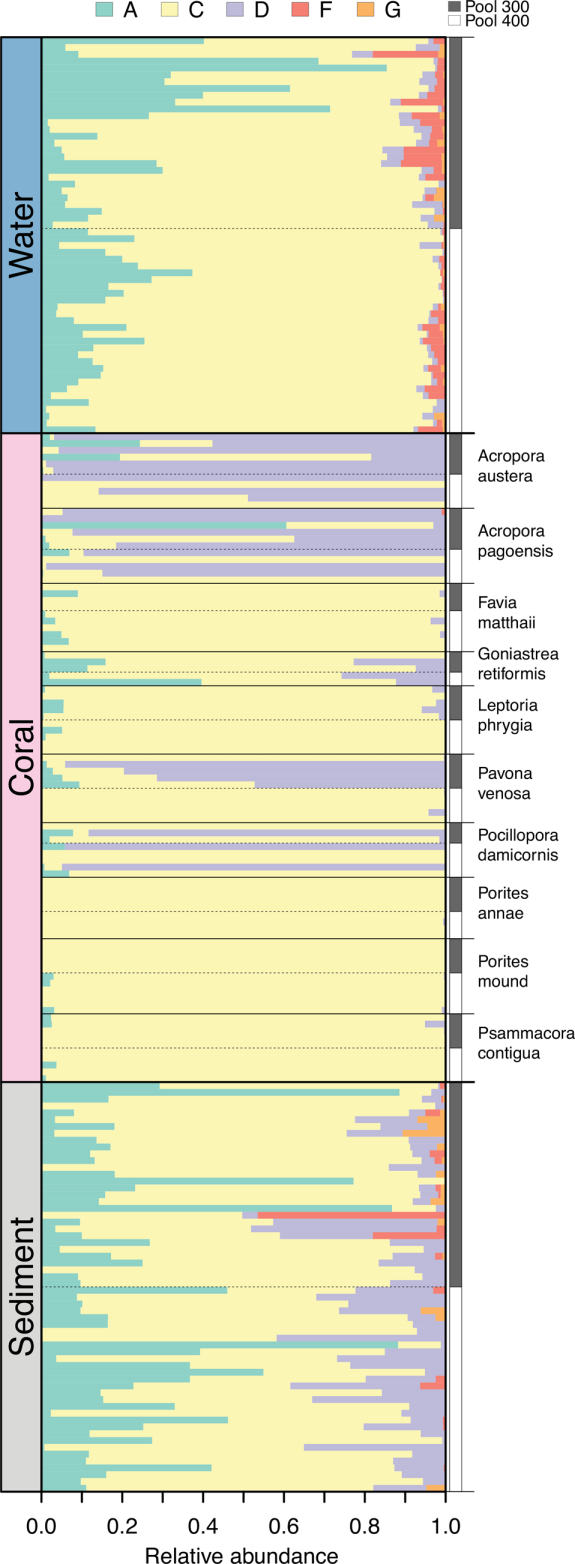
*Symbiodinium* clade composition for complete dataset. Bars represent the proportion of sequences in each sample from each clade.

**Fig 2:**
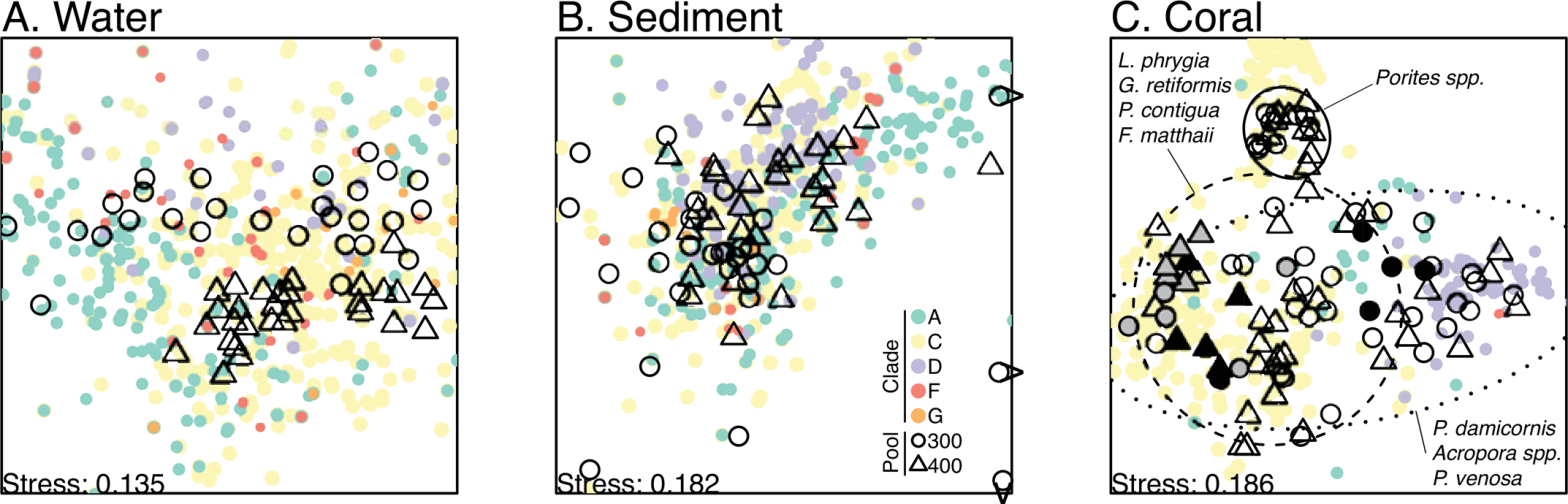
Differentiation of *Symbiodinium* communities between pools. NMDS biplots on Bray-Curtis dissimilarities are shown for water (A), sediment (B), and coral (C). Samples from pool 300 and pool 400 are represented by circles and triangles, respectively. Taxa are plotted in the same ordination space and colored by clade (A = green, C = yellow, D = purple, F = red, G = orange) to show their role in driving the differentiation between samples (e.g., clade D in association with sediment samples from pool 400). Three sediment samples are plotted with arrows indicating they lie outside the plot range. For corals, 90% confidence ellipses surround groups of species categorized qualitatively as low (< 0.4), intermediate (0.4–0.6), or high (> 0.6) overall dissimilarity (Table 1) to illustrate the range of observed variability in different coral species. Coral species whose symbiont communities were significantly different between pools (Table 1) are represented by filled symbols (gray = *Psammacora contigua*, black = *Pavona venosa*).

*Symbiodinium* sequences recovered from corals, sediments, and water included members of clades A, C, D, F, and G. Overall, clade C comprised the highest proportion of sequences (76.5%), followed by clade A (12.5%), clade D (9.9%), clade F (0.9%), and clade G (0.2%). The composition of individual communities varied among compartments (PERMANOVA, p = 0.0001; Fig 1). Water samples were dominated by clades C and A with background F, D, and G, whereas coral samples contained primarily C and D with lesser amounts of clade A. Sediments were dominated by C, A, and D, with individual samples also showing the highest proportions of clades F and G (Fig 1). Beyond these clade-level patterns, each compartment and coral species contained distinct assemblages of sub-clade types (visualized by rank-abundance plots) that ranged from lower (e.g., *Porites* spp.) to higher (e.g., water, sediments) diversity (S2 Fig). Furthermore, and the focus here, communities differed among samples within each compartment and species. The overall dissimilarity (i.e., variability or beta diversity) in *Symbiodinium* composition among samples within a compartment was higher in sediments (0.620) than in water (0.501), while corals, which had the highest dissimilarity when grouped together (0.761), displayed a range of high (>0.6; e.g., *Pocillopora damicornis*, *Pavona venosa*, *Acropora* spp.) to low (<0.4; e.g., *Porites* spp.) variability within individual species (Table 1; Fig 2).

**Table 1:** Bray-Curtis dissimilarities and PERMANOVA tests for effect of pool on community composition. Within-pool dissimilarities are weighted averages of the two pools. Bold text indicates statistically significant results.

Type / Species	n	Average Bray-Curtis dissimilarity			Pool effect PERMANOVA	
		Overall	Within-pool	Between-pool	R^2^	p-value
Water	58	0.5007	0.4785	0.5222	0.0916	**0.0001**
Sediment	60	0.6205	0.6051	0.6354	0.0616	**0.0001**
Coral	95	0.7614	0.7584	0.7643	0.0161	0.1484
* Pocillopora damicornis*	8	0.6675	0.7122	0.6288	0.0393	0.8382
* Pavona venosa*	10	0.6438	0.5500	0.7188	0.3385	**0.0471**
* Acropora austera*	11	0.6220	0.5692	0.6660	0.2327	0.0586
* Acropora pagoensis*	11	0.6192	0.6042	0.6316	0.1308	0.2361
* Goniastrea retiformis*	5	0.5882	0.5669	0.6024	0.3133	0.2083
* Favia matthaii*	10	0.5724	0.5590	0.5841	0.1073	0.4230
* Leptoria phrygia*	10	0.5185	0.5174	0.5193	0.1161	0.3772
* Psammocora contigua*	10	0.5142	0.4691	0.5503	0.2288	**0.0267**
* Porites* mound sp	11	0.3525	0.3603	0.3461	0.0842	0.4728
* Porites annae*	9	0.3112	0.2750	0.3401	0.1986	0.1272

The degree to which this variability could be explained by pool differed among compartments and coral species (Table 1). Visualization of the effect of pool on community composition using NMDS (Fig 2) reveals near complete separation by pool in the water column, some separation and overlap in sediments, and variable levels of separation in different coral species. Positions of *Symbiodinium* taxa in the ordination space reveal which clades are driving the clustering among samples, the most conspicuous being clade D in association with water in pool 300, sediments in pool 400, and *Pavona venosa* in pool 300 (Fig 2). Statistical tests confirmed that symbiont communities were indeed different between pools in both water (PERMANOVA; p = 0.0001) and sediments (p = 0.0001), as well as in the corals *Psammacora contigua* (p = 0.0301) and *Pavona venosa* (p = 0.0479), but not in the other eight coral species (p>0.05; Table 1). In compartments with a significant pool effect, the effect sizes were generally low (R^2^ = 0.06, 0.09 for sediments and water; 0.23, 0.34 for *P*. *contigua* and *P*. *venosa*), indicating that most of the variability occurred within rather than between pools.

### Differential abundance between pools

**Fig 3:**
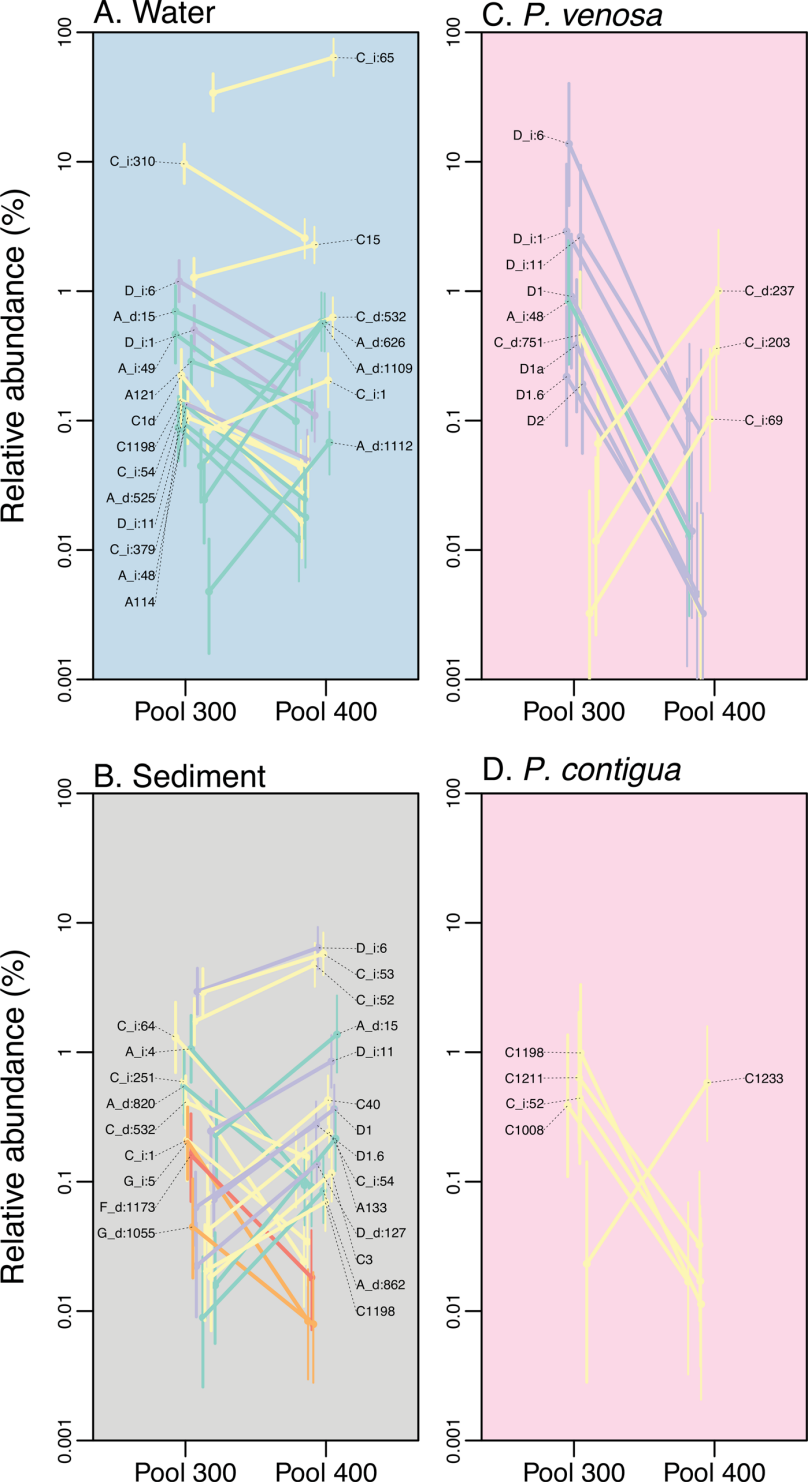
Differential abundance of *Symbiodinium* taxa between pools. Connected points colored by clade (A = green, C = yellow, D = purple, F = red, G = orange) indicate the relative abundance in each pool (computed relative to a “sum-OTU” with 95% credible limits [16]) of all differentially abundant taxa in water (A), sediment (B), *Pavona venosa* (C), and *Psammocora contigua* (D).

Identification of differentially abundant taxa (i.e., more abundant in one pool or the other) following a significant PERMANOVA result revealed more widespread differences in water (21 taxa comprising 60% of water sequences; Fig 3A) and sediments (23 taxa, 52%; Fig 3B) compared to *Pavona venosa* (12 taxa, 16%; Fig 3C) and *Psammocora contigua* (5 taxa, 1.5%; Fig 3D). In water, C15- and C17-like types were less abundant in pool 300, while C1- and C3-like types were more abundant. Clade D types (similar to D1 and D4) were also more abundant in pool 300 and several clade A taxa were more (A2- and A3b-like) or less (A3- and strain HA3-5-like) abundant (Fig 3A). In sediments, certain clade A (similar to A1 and A2), C (similar to C15, C17, C62, and C91), F, and G types were more abundant in pool 300, while other clade A (similar to free-living strain HA-3-5) and C types (similar to C1, C3, and C40) as well as clade D types (similar to D1, D2, and D4), were less abundant (Fig 3B). *Pavona venosa* contained near hundredfold higher levels of several clade D types (similar to D1, D2, and D4) as well as a C15- and A3b-like type in pool 300 (the largest fold-changes observed anywhere), along with lower levels of other C1-, C3-, and C15-like types (Fig 3C). *Psammocora contigua* hosted more of C1 and C3-like types in pool 300, and less of a C15-like type, though these taxa each comprised <1% of the community (Fig 3D).

**Fig 4:**
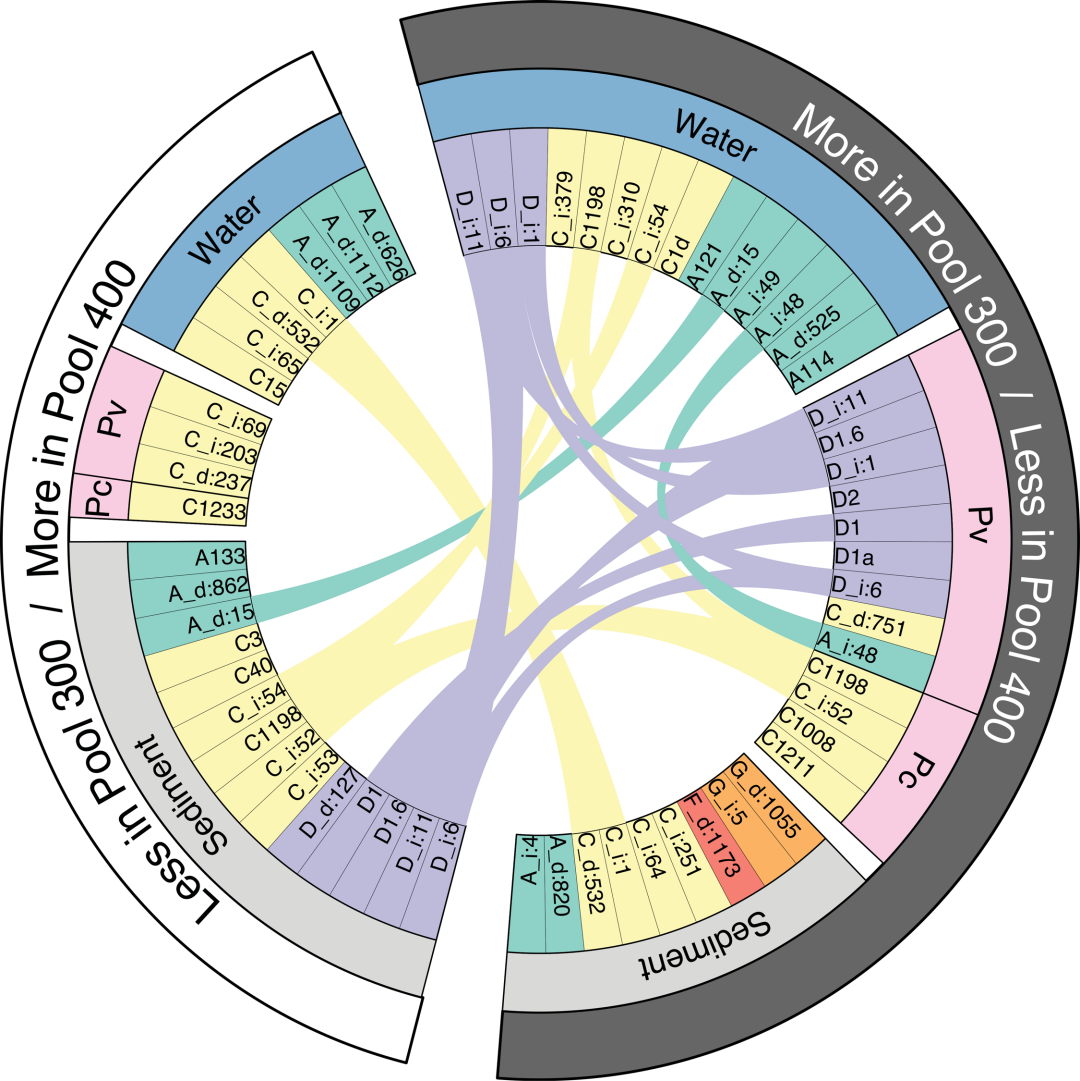
Altered compartmental distributions of *Symbiodinium* between pools. The inner ring shows *Symbiodinium* taxa (clade A = green, C = yellow, D = purple, F = red, G = orange) that were differentially abundant in each compartment (middle ring: water = blue, sediment = gray, coral = pink, *Pavona venosa* = Pv, *Psammocora contigua* = Pc) between pools. The outer ring indicates whether taxa were more or less abundant in each pool, and ribbons connect instances where taxa were differentially abundant in multiple compartments. Ribbons reveal different compartmental distributions of taxa in each pool: in pool 300 (vs. 400), 8 taxa (including 4 clade D) were less abundant in sediments and more abundant in water and/or coral, while 2 other clade C taxa were more abundant in sediments and less abundant in water.

Analyzing between-pool differences in each compartment revealed that many taxa that were less abundant in one compartment were also more abundant in another (Fig 4), indicating that the compartmental distribution of these taxa is altered between pools. For example, of the 14 taxa that were less abundant in the sediments of pool 300 (vs. pool 400), 8 of these were also more abundant in the water and/or coral, including 4 out of 5 clade D types. Likewise, 2 out of 4 clade C types that were less abundant in the sediments of pool 400 were more abundant in water.

### Differences within pools

**Fig 5:**
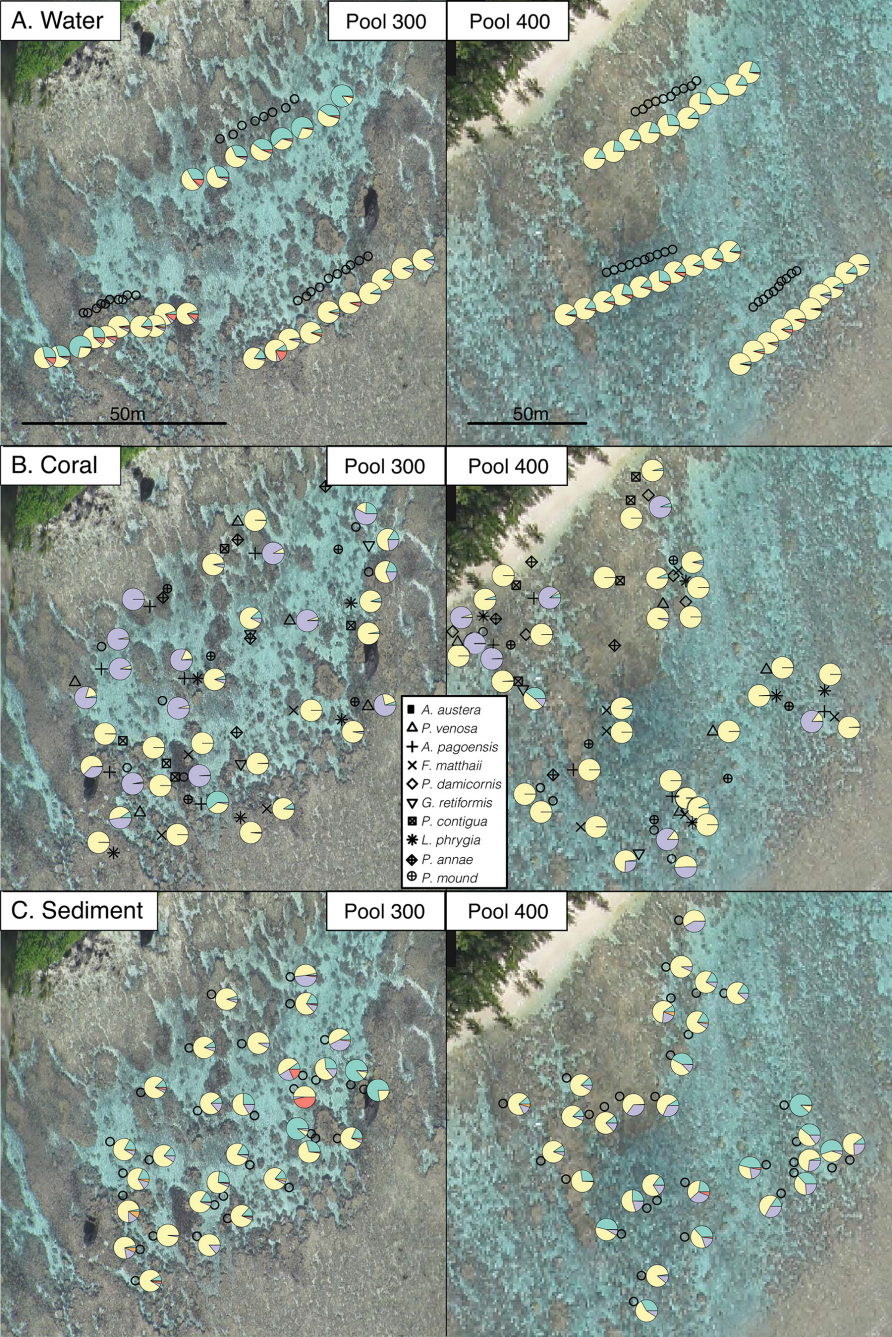
Location and *Symbiodinium* clade composition of water, coral, and sediment samples in each pool. Points represent sample positions and pie charts represent clade composition (A = green, C = yellow, D = purple, F = red, G = orange). Water samples (A) showed more clade A near shore, while sediments (C) showed more clade A further from shore, and clusters of more similar communities (e.g., clades D and F in the northeast and clade G in the southwest corners of pool 300; similar mixtures of C, D, and A in northwest corner of pool 400). Corals (B) tended to have more clade D near pool edges, especially in pool 400. Clade composition for *Porites* spp. (i.e., species with the lowest dissimilarity, Table 1) is not shown here, but can be seen in Fig 1. While data are visualized at the clade level, spatial autocorrelation tests reflect patterns in total diversity.

Within-pool variation in *Symbiodinium* assemblages was investigated for each compartment and coral species by visual mapping and Mantel tests for spatial autocorrelation. Rescaling the spatial extents of sample positions between pools did not affect the results of Mantel tests. Water samples showed significant spatial autocorrelation in both pools (p = 0.001), with visual mapping of symbiont taxa (aggregated to the clade level) revealing clear differences among transects and generally higher abundances of clade A further inshore (Fig 5). Spatial autocorrelation was also detected in sediments in pool 400 (p = 0.001) but not in pool 300 (p = 0.183). No spatial autocorrelation was detected in individual coral species (p>0.05 for all tests), likely due to small sample sizes (n = 2–6 per species per pool). However, visual mapping shows potential trends of higher abundance of clades A and D in corals near pool edges (Fig 5) and high average Bray-Curtis dissimilarities within pools for most coral species (Table 1) imply that spatial structure may exist even though it was not statistically detectable.

## Discussion

We investigated differences in symbiotic and free-living *Symbiodinium* communities between two backreef pools with contrasting thermal environments. While mean temperatures in the pools were similar (300: 29.0°C, 400: 29.1°C), the smaller pool 300 experienced higher maxima (34.3°C vs. 33.3°C) and a greater temperature range (7.7°C vs. 5.9°C) during the 2-week study period (Yost et al., submitted), which is consistent with long-term temperature patterns recorded in these pools [35]. The pools had strongly and moderately differentiated *Symbiodinium* communities in the water column and sediments, respectively, and coral-associated communities showed between-pool differences in two out of ten species (Figs 1 and 2).

Our analyses were performed on pyrosequencing read abundances of ITS2 taxonomic units, which do not necessarily reflect organismal abundances due to PCR bias and ITS2 copy number variation among taxa [39,40]. These factors limit our ability to draw conclusions based on the proportions of different taxa within samples; however, they do not directly confound relative comparisons among samples or groups having similar taxa present in dissimilar proportions (e.g., fold-changes and Bray-Curtis dissimilarities between pools). Likewise, while we cannot map ITS2 sequence diversity directly onto biological or functional diversity, we can analyze and discuss taxa in a broader phylogenetic context (e.g., “C15-like types”) to aid interpretation of ecological patterns. With these limitations in mind, we utilize ITS2 pyrosequencing data to understand variability in *Symbiodinium* community structure in these reef waters, sediments, and corals.

*Pavona venosa* symbiont communities differed dramatically between pools, with less clade C and hundredfold more clade D in the warmer, more variable pool (Fig 3), consistent with the well-documented thermotolerance of clade D [53,54] and its tendency to dominate corals in warm and/or marginal environments [55]. Prior work also revealed that clade D more often dominated *Acropora hyacinthus* in pool 300 than pool 400 [35]. While differentially abundant taxa in *P*. *venosa* together comprised 16% of all reads, between-pool differences in *Psammocora contigua* were more subtle (1.5%), with pool 300 colonies hosting slightly less of a C15-like type (C1233) and more of C1- (C1198, C1211, C1008) and C3-like (C_i:52) types, suggesting that subclade diversity is also responsive to the environment. However, the ecological importance of shifts in low abundance background symbionts are equivocal and not well understood. Nevertheless, “pool” accounted for only 23–34% of the overall dissimilarity among colonies in these two species, indicating that most of the variability occurred at the within-pool scale (Table 1). Symbiont communities in the other eight coral species showed no differences between pools, which reflects either 1) low overall variability (i.e., specificity), or 2) high variability at smaller, within-pool spatial scales.

Low overall variability was observed among *Porites* mound sp. and *Porites annae* colonies (Table 1; Fig 2), which were dominated by the same clade C taxa all closely related to C15 (e.g., C_i:65, C_d:751, C1233, C15; S2 Fig), confirming the known highly-specific symbioses formed by *Porites* corals [10]. Symbiont community structure in these species appears to be constrained by host biology and is consequently insensitive to the range of environmental differences between pools. The lack of between-pool differences in the remaining six species, however, can be attributed instead to high within-pool variation that matched or exceeded the variation between pools. For example, in both pools, some colonies of *Pocillopora damicornis* and *Acropora* spp. were dominated by clade C, while others were dominated by clade D. These species, along with *Pavona venosa*, showed the most overall variability in their symbiont communities (Table 1) and were the only ones that were sometimes dominated by clade D (Fig 1). However, this high variability occurred primarily within pools, suggesting these symbioses may be more sensitive to the range of environmental differences occurring within, rather than between, the two pools. For example, clade D tended to be more abundant toward pool edges (Fig 5), where temperature ranges were higher (Yost et al., submitted), although small sample sizes precluded a statistical test of this link. While clade C taxa dominated all colonies of *Favia matthaii*, *Leptoria phrygia*, and *Goniastrea retiformis*, there were variable amounts of clades A and D in colonies in both pools, resulting in intermediate overall dissimilarity primarily at the within-pool scale (Table 1).

The finding that most of the variability among symbiont communities in corals occurred within, rather than between, pools supports previous work showing that symbiont composition in some corals varies more over meters than kilometers (e.g., *Montipora capitata* [56]) and suggests that environmental influences on symbiont assemblages may operate over small spatial scales. While other studies have shown changes in the dominant symbiont type over temperature and light gradients across reefs [12,57,58] and even within colonies [59,60], our whole-community analyses of a set of coral species reveals that variability in community structure (e.g., Table 1) over scales of meters may be common in more coral taxa than previously thought. This variability may be driven by abiotic and biotic factors such as temperature, light, water flow, host genetic identity [56] and ontogeny [61], the surrounding metacommunity composition [32], or stochasticity [62]. Testing these hypotheses will require increased biological sampling and collection of environmental data at high spatial resolution.

By comparison to corals, *Symbiodinium* in seawater and sediments showed stronger patterns of differentiation between pools (Fig 2) and spatial autocorrelation within pools (Fig 5). Higher sample sizes for sediment and water than individual coral species likely increased our power to detect these patterns. However, the significant within-pool structuring of *Symbiodinium* in the water column of both pools was unexpected given that water flow and tidal flushing can cause rapid community homogenization and turnover. Nevertheless, water column communities in both pools were characterized by a higher abundance of clade A taxa nearer to shore. In sediments, stronger spatial autocorrelation may have been detected in pool 400 due to its larger size and therefore greater potential for environmental variation. Nevertheless, clusters of more similar communities were apparent in the sediments of both pools (e.g., clades D and F in the northeast and clade G in the southwest corners of pool 300; similar mixtures of C, D, and A in northwest corner of pool 400), and clade A tended to be more abundant further from shore. However, sediment patterns were less clear than those in the water, possibly reflecting greater microhabitat heterogeneity [63].

Despite the significant variability in free-living *Symbiodinium* communities within pools, many differences were also detected between pools. In pool 300, various clade A and C taxa were more or less abundant in both water and sediments compared to pool 400 (Fig 3), which may reflect environmental niche differentiation among closely-related types within these clades [57]. In contrast, clade D types were consistently less abundant in pool 300 sediments but more abundant in water and coral (vs. pool 400; Figs 3 and 4), suggesting their compartmental distribution may be influenced by the environment. Several clade A and C taxa were also less abundant in one compartment and more abundant in another when comparing the two pools (Fig 4). These patterns allude to an environmental influence on the interactions and connectivity among *Symbiodinium* in different compartments and suggest that the surrounding metacommunity may impact local symbiont assemblages by influencing availability, uptake, and dispersal [32]. In particular, the trend of many taxa in the warmer, more variable pool being less abundant in sediments and more abundant in water and coral suggests that environmental variation and extremes may expand their distribution. While further study is necessary to confirm such linkages, these findings suggest that an environmentally-mediated metacommunity framework [31] may help explain patterns of *Symbiodinium* distribution and abundance on coral reefs.

In conclusion, our findings suggest that symbiont assemblages in different reef habitat compartments and coral species may be influenced to varying degrees by environmental differences between and within these back-reef pools. While *Porites* species exhibit low variability in symbiont composition indicative of highly specific symbioses, the other corals studied here showed intermediate to high variability primarily at within-pool scales, suggesting that responsiveness to environmental variability over small spatial scales may be more common than previously thought. High variability in free-living *Symbiodinium* and altered compartmental distributions of taxa between pools suggest that the reef-scale metacommunity may also play a role in shaping local community structure.

## Supporting Information

S1 Fig Distribution of sequence counts per taxon and per sample in the complete dataset. (PDF)

S2 Fig *Symbiodinium* community composition barplots for each habitat compartment and coral species. Bars colored by clade (A = green, C = yellow, D = purple, F = red, G = orange) represent the proportion of sequences within the compartment comprised by each taxon. Only taxa comprising > 0.1% of all sequences are shown. Taxa that were differentially abundant between pools are indicated by asterisks. In *Acropora austera* and *Acropora pagoensis*, generalized linear mixed modeling identified some differentially abundant taxa, even though PERMANOVA detected no significant effect of pool in these species (Table 1).
(PDF)

S3 Fig Number of *Symbiodinium* sequences obtained from each sample, sorted by species and compartment. (PDF)

S1 Table Contextualization of internal node and *de novo* taxa among reference sequences. For internal nodes (“X_i:X”), the closest leaf taxon and distance to that taxon are given. For de novo taxa (“X_d:X”), the closest BLAST hit from the reference database and associated e-value are given.
(CSV)
