# Supplementary figures and images for: Variability of Symbiodinium Communities in Waters, Sediments, and Corals of Thermally Distinct Reef Pools in American Samoa
Source: PLoS One. 2015 Dec 29;10(12):e0145099. doi: 10.1371/journal.pone.0145099 (PMC4695085; doi:10.1371/journal.pone.0145099)

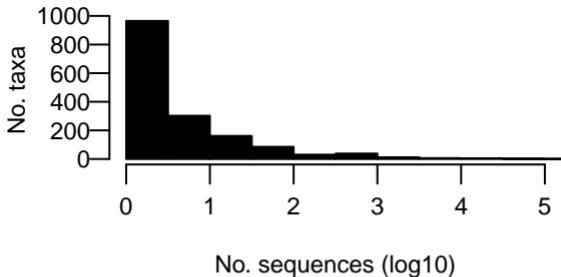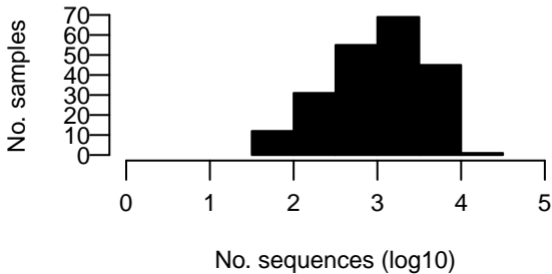

Supplement: S1 Fig — (PDF) [file pone.0145099.s001.pdf]

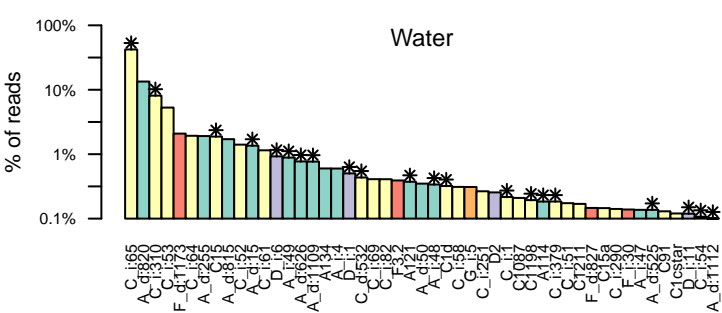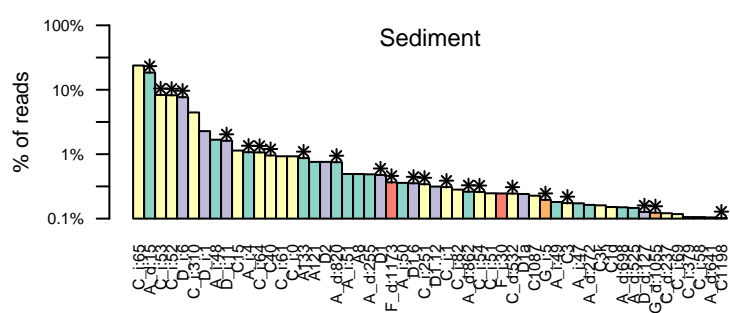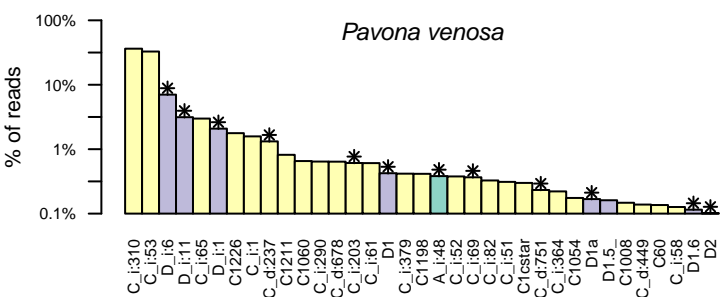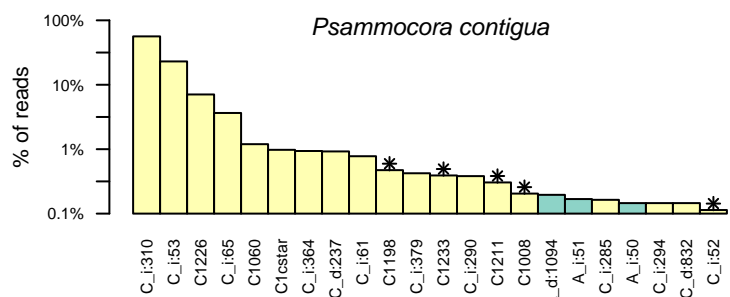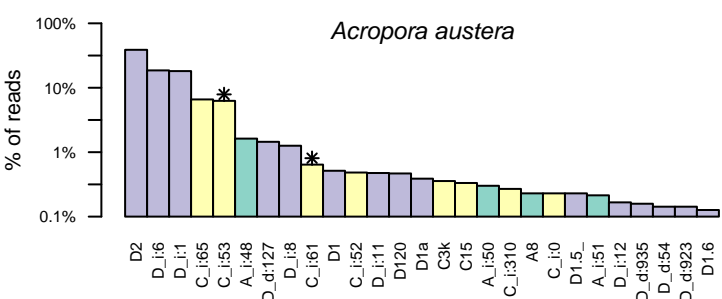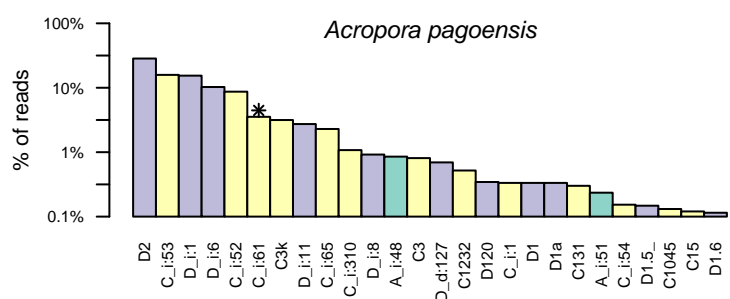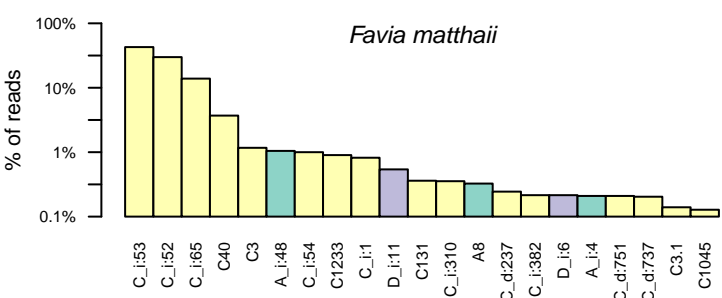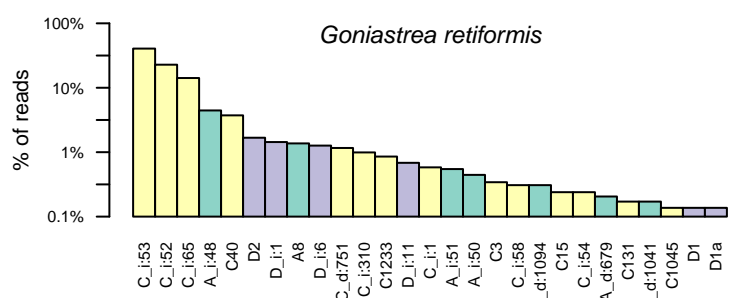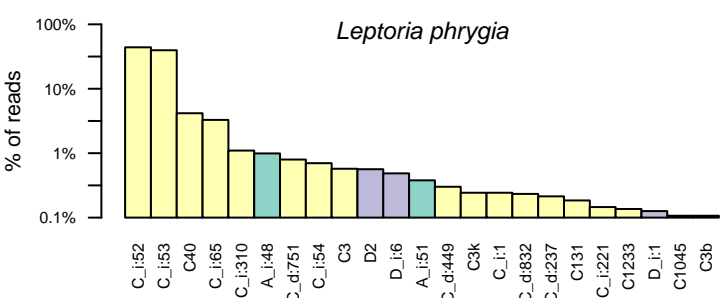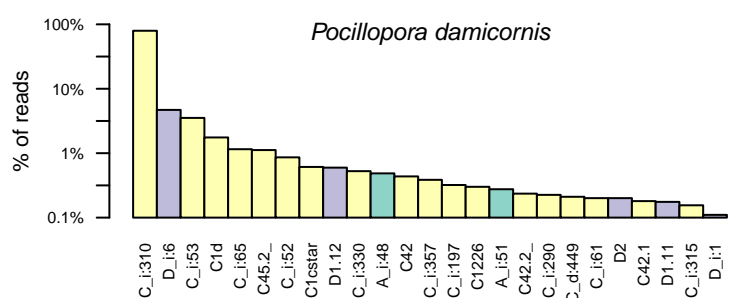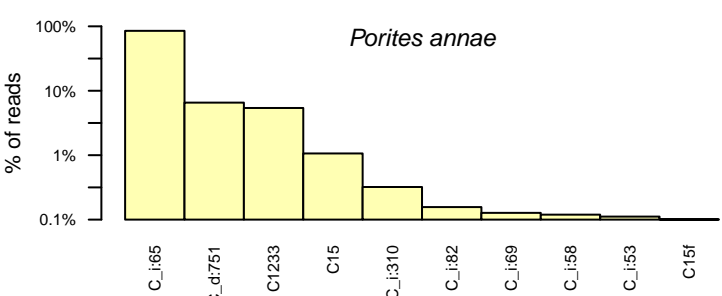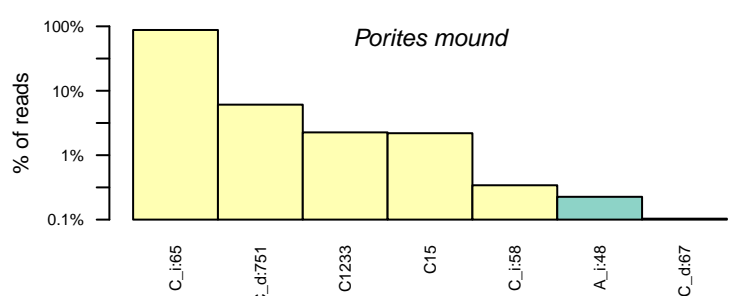

Supplement: S2 Fig — Bars colored by clade (A = green, C = yellow, D = purple, F = red, G = orange) represent the proportion of sequences within the compartment comprised by each taxon. Only taxa comprising > 0.1% of all sequences are shown. Taxa that were differentially abundant between pools are indicated by asterisks. In Acropora austera and Acropora pagoensis, generalized linear mixed modeling identified some differentially abundant taxa, even though PERMANOVA detected no significant effect of pool in these species (Table 1). (PDF) [file pone.0145099.s002.pdf]

log<sub>10</sub> Symbiodinium sequences

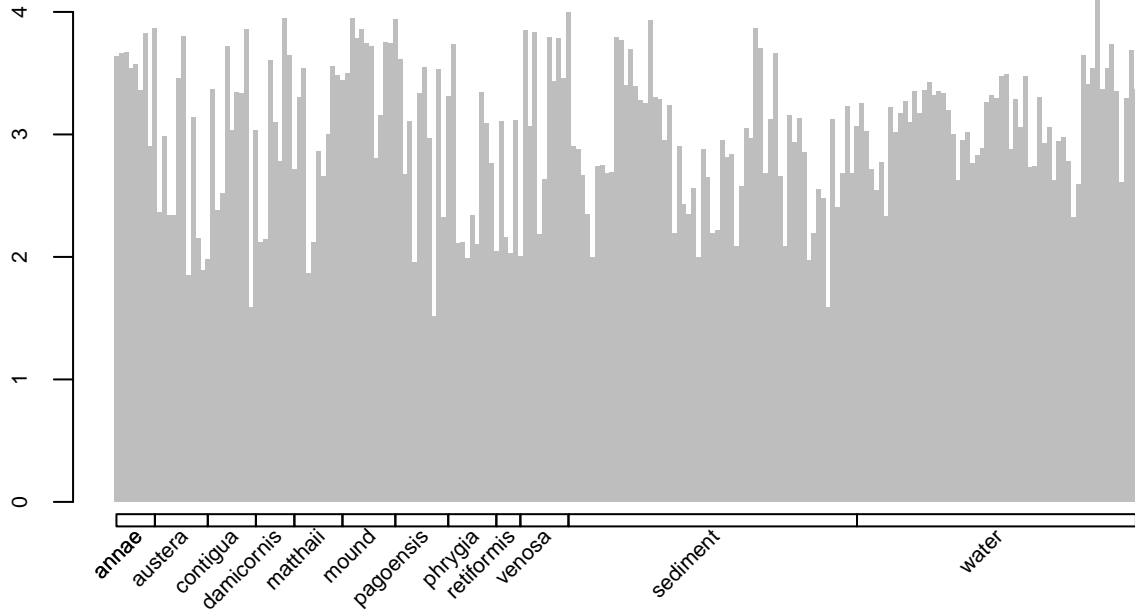

Samples (by species/compartiment)

Supplement: S3 Fig — (PDF) [file pone.0145099.s003.pdf]
